# Incidence of Clinically Documented Phantom Limb Pain During Hospitalization and Preoperative Risk Factors in Patients Who Underwent Nontraumatic Major Lower Limb Amputation: A Single-Center, Retrospective Study

**DOI:** 10.3390/medicina62050848

**Published:** 2026-04-29

**Authors:** Tsutomu Mieda, Hideyuki Asaka, Takumi Yamaguchi, Yohei Kawasaki, Yuta Horikoshi, Tina Nakamura, Asako Tominaga, Hiroshi Hoshijima, Hiroshi Nagasaka, Noritaka Imamachi, Tatsuo Yamamoto

**Affiliations:** 1 Department of Anesthesiology, Saitama Medical University Hospital, 38 Morohongo, Moroyamacho, Irumagun, Saitama 350-0495, Japan; miebenben@gmail.com (T.M.); h.asaka.smu@gmail.com (H.A.); motokensmu@gmail.com (Y.H.); chinakamura86@gmail.com (T.N.); miyasako19830930@gmail.com (A.T.); hhoshi6@gmail.com (H.H.); nkrikoim@gmail.com (N.I.); yamyam3218@gmail.com (T.Y.); 2 Department of Biostatistics, Graduate School of Medicine, Saitama Medical University, 38 Morohongo, Moroyamacho, Irumagun, Saitama 350-0495, Japan; takumi@saitama-med.ac.jp (T.Y.); ykawasaki@saitama-med.ac.jp (Y.K.); 3 Division of Dento-Oral Anesthesiology, School of Dentistry, Tohoku University Graduate, 4-1 Seiryomachi, Aoba, Sendai 980-0872, Japan

**Keywords:** phantom limb pain, nontraumatic lower limb amputation, risk factors

## Abstract

*Background and Objectives:* Phantom limb pain (PLP) frequently occurs after lower limb amputation (LLA). However, a consensus has not been reached regarding its incidence, particularly in nontraumatic amputations, because reported estimates vary according to case mix, follow-up duration, and outcome definitions. Data from Japan remain limited. *Methods:* After approval by the Institutional Review Board, the electronic medical records of patients who underwent above-knee amputation (AKA) or below-knee amputation (BKA) at Saitama Medical University Hospital between 1 January 2012 and 31 December 2024 were retrospectively reviewed. The primary outcome was the incidence of clinically documented PLP during hospitalization, typically within approximately 1–2 months after amputation. PLP was defined as a painful sensation in the amputated limb documented in the medical record and diagnosed by surgeons or anesthesiologists. Postamputation pain due to symptomatic neuroma was not classified as PLP. Patients with only nonpainful phantom limb sensations were not considered to have the primary outcome. Patients aged 18 years or older who required nontraumatic AKA or BKA were included. Patients who (1) died within 30 days of surgery or (2) were unable to communicate were excluded. *Results:* Clinically documented PLP occurred in 31 of 298 patients (10.4%; exact 95% CI, 7.2–14.4%) during hospitalization. In the prespecified primary model including age and preoperative pain, younger age (adjusted odds ratio (OR) 0.96 per 1-year increase, 95% confidence interval (CI) 0.93–0.99; *p* = 0.008) and preoperative pain (adjusted OR 16.34, 95% CI 3.75–71.24; *p* < 0.001) were associated with PLP. In an exploratory model additionally including postoperative pain on the day of surgery, postoperative pain was not independently associated with PLP. Firth penalized logistic regression yielded similar results. *Conclusions:* This study found a 10.4% incidence of clinically documented PLP during hospitalization after nontraumatic major LLA. Younger age and preoperative pain were associated with PLP, although the estimates should be interpreted cautiously because only 31 PLP events occurred.

## 1. Introduction

A review of the literature revealed that the incidence of phantom limb pain (PLP) in adults after lower limb amputation (LLA) is high (17–97%) [1,2,3,4,5,6,7]. Anatomical changes associated with PLP occur at the peripheral, spinal cord, and brain levels, including spinal dorsal horn sensitization and plasticity and short- and long-term alterations at the molecular and topographical levels. The molecular reorganization process in PLP involves excessive activation of *N*-methyl-d-aspartate (NMDA) receptors in the dorsal spinal cord, leading to inflammatory mechanisms at the peripheral and central levels. At the brain level, changes are observed via imaging. However, the mechanisms underlying PLP remain unclear, and researchers and clinicians continue to debate them, as well as the contributions of the central nervous system (CNS) and/or peripheral nervous system [8].

The causes of LLA include chronic limb ischemia, acute limb ischemia, infection, cancer, and trauma [9]. Diabetes mellitus (DM) reportedly increases the risk of peripheral artery disease and infection, which can lead to LLA [10,11]. Therefore, patients with nontraumatic LLA often have comorbidities, and the incidence of PLP has been discussed in these patients [1,2]. Several retrospective cohort studies have revealed that DM is a factor protecting against the development of PLP and symptomatic neuromas [11,12,13]. This result is based on the hypothesis that patients with DM are less likely to develop neuropathic pain because their nerve regeneration potential is suppressed. However, Clark et al. reported no difference in the prevalence of PLP between patients with and without DM or between the duration of diabetes and the prevalence of PLP [14]. Furthermore, patients who undergo LLA due to vascular disease are traditionally considered to experience less PLP than nonvascular leg amputees [15]. Conversely, in a systematic review and meta-analysis, Langeveld reported a higher incidence of PLP after dysvasculitic LLA [15]. Therefore, no consensus is available on the incidence of phantom pain after amputation due to causes other than trauma, as it varies with comorbidities (associated medical diseases) [1,2].

In addition, the incidence of PLP has been reported to vary depending not only on comorbidities but also on the country in which the paper was published [2]. A meta-analysis that stratified studies by country development status suggested that the prevalence of PLP was significantly lower in developing countries than in developed countries (54% vs. 67%) [2]. These results may reflect differences between such countries regarding medical standards, research activities related to PLP, genetic and cultural circumstances, and the success rate of recruiting patients for PLP studies [2,4].

However, most studies on the incidence of and risk factors for PLP have been published in North America and Europe, with few published in Japan; to our knowledge, Noguchi’s study of only 44 patients is the only study published in Japan [12]. In their retrospective study, they reported that PLP occurred in 22 of 44 patients (50%); however, they also reported that it occurred in 7 of 21 DM patients and 5 of 7 trauma patients. The incidence of PLP after nontraumatic amputation may be lower than that reported in previous papers, although this remains uncertain because the sample size is small in Japan.

We therefore retrospectively investigated the incidence of clinically documented PLP during hospitalization (approximately 1–2 months after amputation) and preoperative risk factors for PLP in patients who underwent nontraumatic major LLA at our institution in Japan. We hypothesized that the incidence of clinically documented PLP during hospitalization following nontraumatic amputation might be lower than that previously reported in Japan [12]. The primary outcome of the present study was the incidence of clinically documented PLP during hospitalization. Additionally, we also examined preoperative factors associated with the development of PLP following above-knee amputation (AKA) and below-knee amputation (BKA).

## 2. Materials and Methods

### 2.1. Study Design and Sample

This retrospective cohort study was approved by the Institutional Review Board (IRB: approval number 2025-032) of Saitama Medical University Hospital. This study adhered to the guidelines of the Declaration of Helsinki. All methods were performed in accordance with the relevant guidelines and regulations, and the requirement for study-specific informed consent was waived because of the retrospective design and anonymized data handling. Information about the conduct of the study, including its purpose, was made public, and opportunities for refusal were guaranteed whenever possible. Patients who presented to Saitama Medical University Hospital between 1 January 2012 and 31 December 2024 and who required nontraumatic major LLA, i.e., AKA and BKA, were included. The patients had received one of several methods of anesthesia and were identified by reviewing their electronic records. The inclusion criteria were patients who were over 18 years old and required nontraumatic AKA or BKA. Patients who (1) died within 30 days of surgery or (2) were unable to communicate were excluded. Moreover, for patients with repetitive major lower limb amputations in our hospital, cases other than the first were excluded.

### 2.2. Study Variables

The primary outcome variable was the incidence of clinically documented PLP during hospitalization. PLP was defined as a painful sensation in the amputated limb documented in the medical record and diagnosed by surgeons or anesthesiologists within 1–2 months after amputation. Postamputation pain due to symptomatic neuroma was not classified as PLP. PLP was ascertained by a retrospective review of the electronic medical records using this chart-based definition. Pain documented only as residual limb pain, stump pain, or wound pain, without pain perceived in the amputated limb, was not classified as PLP. If the chart description was insufficient to distinguish PLP from other postoperative pain syndromes, the case was not classified as PLP. In our hospital, almost all patients were discharged within 1–2 months, and most were transferred to a rehabilitation hospital. Because detailed post-discharge data were unavailable owing to the retrospective nature of the study, the observation period was limited to hospitalization (approximately 1–2 months). Patients with only phantom limb sensations, which are nonpainful sensations in the amputated limb, were not considered to have the primary outcome. When a patient complained of pain but was unable to describe it clearly, they were excluded because communication was not possible. Although the diagnosis of PLP was based on chart documentation, some misclassification between PLP and other postoperative pain syndromes could not be completely excluded.

### 2.3. Anesthesia and Perioperative Management

The protocol for anesthesia management was not fixed, and anesthetic decisions were based on the anesthesiologist’s preference. Analgesics, such as opioids, nonsteroidal anti-inflammatory drugs (NSAIDs), tramadol, pregabalin, and/or mirogabalin, were administered perioperatively according to the patient’s needs, as determined by the anesthesiologists or ward surgeons. Ketamine was not used in this cohort. Thus, various types of anesthesia, such as general, regional, and spinal anesthesia, were chosen according to the patient’s condition. Under general anesthesia, patients were administered total intravenous or inhalational anesthesia.

### 2.4. Data Collection

The data collected from the electronic medical records included age, sex, body mass index (BMI), American Society of Anesthesiologists Physical Status (ASA PS), smoking status within 1 month before surgery, history of diabetes mellitus (DM), dialysis, history of ischemic heart disease, history of cerebrovascular disease, hypertension requiring medication, steroid use, preoperative insulin use, the presence of moderate-to-severe preoperative pain on the day of surgery (preoperative pain), the presence of moderate-to-severe postoperative pain on the day of surgery (postoperative pain), preoperative serum albumin levels, bilateral amputations, conversion from BKA to AKA, whether the amputation was an emergency, the presence of ischemic limbs due to vascular disorders, surgery type, anesthesia type, and perioperative analgesic use. Pregabalin and mirogabalin were considered neuropathic pain drugs. Because this study included surgical patients over a 13-year period beginning in 2012 and the numerical rating scale (NRS) was not consistently used to assess pain on the day of surgery until approximately 2015, pain assessment methods were not uniform across the study period. Therefore, for analysis, pain on the day of surgery was harmonized as a binary variable indicating the presence or absence of moderate-to-severe pain; mild pain was not included in the preoperative or postoperative pain exposure variables. Bilateral amputations were defined as bilateral amputations at the time of initial amputation surgery. Regarding cases where major amputation was first performed at another hospital and then at our hospital for the second time, those where the amputation level was changed from first BKA in another hospital to second AKA at our hospital (same-side AKA after BKA) were counted as one case (i.e., conversion to AKA after BKA), and contralateral amputations performed at a later date were also counted as one case (i.e., contralateral BKA or AKA). If a single patient underwent multiple amputations, the first recorded case in our hospital was used as a variable. Therefore, one patient corresponds to one procedure. Each case was included as a related variable.

### 2.5. Statistical Analysis

Continuous variables are presented as means ± standard deviations (SDs). Univariate comparisons between patients with and without PLP were performed descriptively using Student’s *t* test for continuous variables and Fisher’s exact test for categorical data. These univariate comparisons were not used for variable selection. Because only 31 PLP events occurred, the multivariable analysis was kept small. The prespecified primary model included age and preoperative pain as clinically selected preoperative covariates. A separate exploratory model additionally included postoperative pain on the day of surgery. Complete-case logistic regression was used for each model, and Firth penalized logistic regression was performed as a sensitivity analysis for sparse-event bias. The incidence of clinically documented PLP during hospitalization is reported with an exact 95% confidence interval. Age was evaluated per 1-year increase, and the odds ratio per 10-year increase was also calculated and reported. All statistical analyses were performed using R software (version 4.5.2; R Foundation for Statistical Computing, Vienna, Austria), with a significance level (*p* value) of 0.05 for all analyses.

## 3. Results

**Figure 1:**
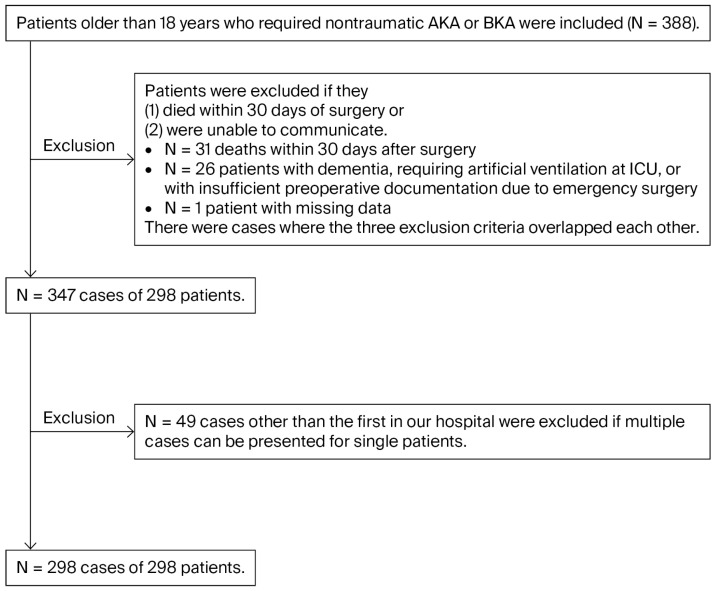
Study flow diagram.

A total of 388 cases were screened, and the final sample size was 298 cases across 298 patients (Figure 1). The mean age was 68.9 ± 13.3 years, and 86 patients (28.9%) were female (Table 1). Clinically documented PLP occurred in 31 of the 298 patients during hospitalization (10.4%; exact 95% CI, 7.2–14.4%). Overall, 30 of the 31 PLP cases were documented as mild, and only 1 case was severe. Ketamine was not used in this cohort. Given that neither epidural anesthesia nor peripheral nerve block with a continuous catheter was implemented in any patient, these anesthetic techniques were not included as variables. There were three cases of bilateral amputation, and none of them resulted in PLP.

Univariable analyses are presented as descriptive comparisons and revealed statistically significant differences in age (*p* = 0.022), preoperative pain (*p* < 0.001), and postoperative pain (*p* < 0.001) between patients with and without PLP. In the prespecified primary model including age and preoperative pain, younger age (OR 0.96, 95% CI 0.93–0.99; *p* = 0.008) and preoperative pain (OR 16.34, 95% CI 3.75–71.24; *p* < 0.001) were associated with PLP (Table 2). In the exploratory model additionally including postoperative pain on the day of surgery, age (OR 0.96, 95% CI 0.93–0.99; *p* = 0.008) and preoperative pain (OR 13.61, 95% CI 1.53–121.26; *p* = 0.019) remained associated with PLP, whereas postoperative pain was not associated with PLP (OR 1.28, 95% CI 0.14–11.67; *p* = 0.830). The OR per 10-year increase in age was 0.67 in both models. In the corresponding Firth models, the primary model yielded ORs of 0.96 (95% CI 0.93–0.99; *p* = 0.008) for age and 13.03 (95% CI 4.12–65.65; *p* < 0.001) for preoperative pain. In the exploratory Firth model, the ORs were 0.96 (95% CI 0.93–0.99; *p* = 0.008), 10.24 (95% CI 1.75–111.22; *p* = 0.006), and 1.22 (95% CI 0.15–11.07; *p* = 0.860) for age, preoperative pain, and postoperative pain, respectively. The crude incidence of PLP was 17.7% (29/164) in patients with preoperative pain and 1.5% (2/134) in those without preoperative pain.

## 4. Discussion

The 10.4% estimate in this study should be interpreted as the incidence of clinically documented PLP during hospitalization, typically within approximately 1–2 months after amputation, rather than the overall incidence of PLP after amputation. This finding is consistent with our hypothesis that the incidence of clinically documented PLP during hospitalization following nontraumatic amputation might be lower than that previously reported [12] because we employed a medical records review approach similar to that used by Noguchi et al. [12] as the data collection method. In a retrospective cohort study based on large-scale population-based databases, the incidence rate of PLP after amputation, including that of fingers and toes, was 4.8%, as reported by Cho et al. in Korea [16]. Moreover, in a retrospective study conducted in China using telephone interviews, Yin et al. reported that PLP was observed in 29% of amputees [4]. This study involved 391 patients, 80% of whom underwent major LLA.

However, the abovementioned reports are retrospective studies, and direct comparisons should be made cautiously because follow-up duration, methods of pain ascertainment, and outcome definitions differed substantially across studies. Although the outcomes of our study, which were limited to the period within 1–2 months postoperatively, are consistent with those reported by Noguchi et al. [12], other PLP studies varied in their reporting of outcomes, with some failing to clearly document them [16]. Therefore, the lower incidence observed in our cohort should not be attributed to racial or regional factors alone. Prospective studies should be conducted in Japan to investigate the incidence of PLP.

Various previous studies have reported early onset of PLP within a few days after surgery in most patients, regardless of the indication for amputation [17,18,19,20,21]. However, because follow-up in our study was limited to hospitalization, PLP developing after discharge may have been missed. Most PLP cases in our cohort were mild during hospitalization. Therefore, the present findings may be more informative for early clinically documented PLP than for persistent or severe long-term PLP. Preoperative pain showed not only a large adjusted odds ratio but also a clinically meaningful absolute risk difference, with PLP occurring in 17.7% of patients with preoperative pain and in 1.5% of those without preoperative pain.

However, reports concerning the association between preoperative pain and PLP are conflicting [2]. Studies of cohorts with peripheral vascular disease and tumors have also reported that preoperative pain does not affect the incidence of PLP [22], while most studies support the finding that amputees who experience pain prior to amputation are more likely to develop PLP [2].

This study also considered the relationship between age and the incidence of PLP in patients who underwent nontraumatic amputation. In another study, Ahmed et al. examined the incidence of PLP and independent factors associated with LLA due to malignancy in patients with a mean age of 38 years [18]. Their study revealed that the incidence of PLP was 42%, and it did not increase with age. Lans et al. investigated the incidence of PLP and its independent factors in patients with an average age of 60 years who underwent LLA, 90% of whom had nontraumatic LLA and 10% of whom had traumatic LLA. Their study revealed that the incidence of PLP was 34%, and it decreased with age [11]. Thus, in the present study, which included patients with a mean age of 69 years, the odds of PLP were lower with increasing age, similar to the findings reported by Lans et al. [11]. Physiological studies consistently indicate that a decrease in NMDA receptor function with age is likely to influence the induction of α-amino-3-hydroxy-5-methyl-4-isoxazole propionic acid (AMPA) receptor-mediated synaptic plasticity [23,24]. Our findings are compatible with the above physiological studies.

The presence of DM, as described in the Background Section, did not significantly influence the development of PLP in this study. As noted by Clark et al., we did not examine the severity of DM neuropathy in our patients, and thus, our result may not represent a strict comparison of the degree of peripheral neuropathy and the degree of PLP [14]. Therefore, in future studies, a comparison of the PLP between patients with and without DM should include a rigorous comparison of peripheral neuropathy.

Although controversy exists about the incidence of PLP after the amputation of ischemic limbs due to vascular disorders, as discussed in the Background Section [15], in our study, no significant difference was observed in the incidence of PLP in patients with or without an ischemic limb due to vascular disease. The presence of preoperative pain may be more important for the incidence of PLP because amputees with vascular causes who experience severe pain in the limb prior to amputation have been reported to be more likely to experience PLP [21]. This topic requires further study.

In our present study, the incidence of clinically documented PLP during hospitalization following nontraumatic LLA was 10.4%, which is lower than that reported in previous studies [1,2,3,4,5,6,7,12]. Furthermore, the severity of pain was mostly mild. As our findings are limited to the 1–2 months postoperatively, they cannot be directly compared with those of reports involving long-term follow-up. However, there was a case of severe PLP that was difficult to treat for pain in our present study. Therefore, we believe that PLP should not be overlooked and should continue to be monitored closely after nontraumatic LLA, as we have done thus far, even if the incidence is low and the severity of pain is mild, as in our present study.

This study has several limitations. First, it was a retrospective, single-center study, and follow-up was limited to the hospitalization period; therefore, PLP developing after discharge may have been missed. Second, the outcome was determined by chart review, and some misclassification between PLP and other postoperative pain syndromes, including residual limb pain or wound pain, cannot be completely excluded. Third, because only 31 PLP events occurred, the precision of the adjusted estimates was limited, as reflected by the wide confidence intervals, particularly for preoperative pain. Fourth, the reasons for amputation were clinically complex, and we therefore used a simplified classification based on ischemic limbs due to vascular disorders versus other causes. Finally, we did not evaluate longitudinal changes in PLP or its psychological consequences after discharge.

## 5. Conclusions

This study found a 10.4% incidence of clinically documented PLP during hospitalization after nontraumatic major LLA. Younger age and the presence of preoperative pain were associated with PLP, although these estimates should be interpreted cautiously because of the limited number of events and the retrospective chart-based outcome ascertainment. Further prospective studies are necessary to confirm the incidence of and factors affecting PLP.

## Figures and Tables

**Table 1:** Summary of the descriptive statistics and bivariate analysis results for related factors in patients stratified by clinically documented phantom limb pain status during hospitalization (*n* = 298).

	Overall (*n* = 298)	Phantom Limb Pain (*n* = 31)	No Phantom Limb Pain (*n* = 267)	*p* Value
Variables				
Age in years, mean (SD)	68.9 (13.3)	63.7 (14.4)	69.5 (13.1)	0.022 ^#,^*
Sex (female), *n* (%)	86 (28.9)	7 (22.6)	79 (29.6)	0.531 ^$^
BMI in kg/m^2^, mean (SD)	21.3 (4.5)	22.1 (4.8)	21.2 (4.5)	0.297 ^#^
Preoperative serum albumin, mean (SD)	2.3 (0.6)	2.5 (0.6)	2.3 (0.6)	0.178 ^#^
ASA PS (PS2/PS3/PS4), *n* (%)	43 (14.4)/238 (79.9)/ 17 (5.7)	7 (22.6)/23 (74.2)/ 1 (3.2)	36 (13.5)/215 (80.5)/ 16 (6.0)	0.350 ^$^
Smoking status within 1 month before surgery, *n* (%)	45 (15.1)	5 (16.1)	40 (15.0)	0.795 ^$^
Diabetes mellitus, *n* (%)	217 (72.8)	22 (71.0)	195 (73.0)	0.832 ^$^
Dialysis, *n* (%)	133 (44.6)	11 (35.5)	122 (45.7)	0.341 ^$^
History of ischemic heart disease, *n* (%)	93 (31.2)	11 (35.5)	82 (30.7)	0.682 ^$^
History of cerebrovascular disease, *n* (%)	98 (32.9)	7 (22.6)	91 (34.1)	0.230 ^$^
Hypertension requiring medication, *n* (%)	172 (57.7)	15 (48.4)	157 (58.8)	0.337 ^$^
Preoperative steroid use, *n* (%)	23 (7.7)	2 (6.5)	21 (7.9)	1 ^$^
Preoperative insulin use, *n* (%)	152 (51.0)	15 (48.4)	137 (51.3)	0.850 ^$^
Preoperative pain on the day of surgery, *n* (%)	164 (55.0)	29 (93.5)	135 (50.6)	<0.001 ^$,^*
Postoperative pain on day 0, *n* (%)	186 (62.4)	29 (93.5)	157 (58.8)	<0.001 ^$,^*
Conversion to AKA after BKA, *n* (%)	11 (3.7)	0	11 (4.1)	0.612 ^$^
Contralateral BK, *n* (%)	3 (1.0)	0	3 (1.1)	1 ^$^
Contralateral AKA, *n* (%)	12 (4.0)	0	12 (4.5)	0.621 ^$^
Clinical Characteristics				
Emergency case, *n* (%)	59 (19.8)	4 (12.9)	55 (20.6)	0.474 ^$^
Indication for amputation (vascular disor der), *n* (%)	213 (71.5)	23 (74.2)	190 (71.2)	0.835 ^$^
Surgery type (AKA), *n* (%)	111 (37.2)	9 (29.0)	102 (38.2)	0.433 ^$^
Anesthesia type				
General anesthesia alone, *n* (%)	232 (77.9)	22 (71.0)	210 (78.7)	0.361 ^$^
Regional block plus either general anesthesia or sedation, *n* (%)	51 (17.1)	8 (25.8)	43 (16.1)	0.205 ^$^
Spinal block plus sedation, *n* (%)	15 (5.0)	1 (3.2)	14 (5.2)	1 ^$^
Preoperative analgesics				
Opioid use, *n* (%)	13 (4.4)	3 (9.7)	10 (3.7)	0.146 ^$^
Tramadol use, *n* (%)	59 (19.8)	7 (22.6)	52 (19.5)	0.644 ^$^
NSAID use, *n* (%)	69 (23.2)	9 (29.0)	60 (22.5)	0.501 ^$^
Acetaminophen use, *n* (%)	39 (13.1)	2 (6.5)	37 (13.9)	0.399 ^$^
Neuropathic pain drugs, *n* (%)	11 (3.7)	3 (9.7)	8 (3.0)	0.098 ^$^
Intraoperative analgesics				
Opioid use, *n* (%)	256 (85.9)	24 (77.4)	232 (86.9)	0.095 ^$^
NSAID use, *n* (%)	20 (6.7)	2 (6.5)	18 (6.7)	1 ^$^
Acetaminophen use, *n* (%)	121 (40.6)	16 (51.6)	105 (39.3)	0.248 ^$^
Postoperative analgesics				
Opioid use, *n* (%)	73 (24.5)	10 (32.3)	63 (23.6)	0.378 ^$^
Tramadol use, *n* (%)	80 (26.8)	12 (38.7)	68 (25.5)	0.138 ^$^
NSAID use, *n* (%)	43 (14.4)	2 (6.5)	41 (15.4)	0.277 ^$^
Acetaminophen use, *n* (%)	48 (16.1)	4 (12.9)	44 (16.5)	0.797 ^$^
Neuropathic pain drugs, *n* (%)	22 (7.4)	5 (16.1)	17 (6.4)	0.068 ^$^

SD, standard deviation; BMI, body mass index; ASA PS, American Society of Anesthesiologists Physical Status; vascular disorder, ischemic limb due to vascular disorder; AKA, above-knee amputation; BKA, below-knee amputation; NSAID, nonsteroidal anti-inflammatory drug. Preoperative and postoperative pain indicate moderate-to-severe pain documented on the day of surgery. Neuropathic pain drugs refer to pregabalin or mirogabalin. Ketamine was not used in this cohort. * indicates *p* < 0.05. ^#^ Student’s *t* test. ^$^ Fisher’s exact test.

**Table 2:** Primary and exploratory logistic regression analyses for clinically documented phantom limb pain during hospitalization.

Variable	Primary Model	Exploratory Model
	OR (95% CI); *p* Value	OR (95% CI); *p* Value
Age in years	0.96 (0.93–0.99); 0.008 *	0.96 (0.93–0.99); 0.008 *
Preoperative pain (y/n)	16.34 (3.75–71.24); <0.001 *	13.61 (1.53–121.26); 0.019 *
Postoperative pain on the day of surgery (y/n)	—	1.28 (0.14–11.67); 0.830

Primary model: age + preoperative pain. Exploratory model: age + preoperative pain + postoperative pain on the day of surgery, OR: odds ratio; CI: confidence interval. Firth sensitivity analysis results are reported in the Section 3. * indicates *p* < 0.05.

## Data Availability

Data and materials are available from the corresponding author upon reasonable request.

## Abbreviations

The following abbreviations are used in this manuscript:

PLP	phantom limb pain
LLA	lower limb amputation
CNS	central nervous system
OR	odds ratio
CI	confidence interval
AKA	above-knee amputation
BKA	below-knee amputation
BMI	body mass index
ASA PS	American Society of Anesthesiologists Physical Status
NSAID	nonsteroidal anti-inflammatory drug
DM	diabetes mellitus
NRS	numerical rating scale
SD	standard deviation
NMDA	*N*-methyl-d-aspartate
AMPA	α-amino-3-hydroxy-5-methyl-4-isoxazole propionic acid
SOAP	subjective, objective, assessment, and plan
